# Molecular imaging using an anti-human tissue factor monoclonal antibody in an orthotopic glioma xenograft model

**DOI:** 10.1038/s41598-017-12563-5

**Published:** 2017-09-26

**Authors:** Hiroki Takashima, Atsushi B. Tsuji, Tsuneo Saga, Masahiro Yasunaga, Yoshikatsu Koga, Jun-ichiro Kuroda, Shigetoshi Yano, Jun-ichi Kuratsu, Yasuhiro Matsumura

**Affiliations:** 1 Division of Developmental Therapeutics, Exploratory Oncology Research & Clinical Trial Center, National Cancer Center, 6-5-1 Kashiwanoha, Kashiwa, Chiba 277-8577 Japan; 2 Department of Neurosurgery, Faculty of Life Sciences, Kumamoto University, 1-1-1 Honjo, Chuo-ku, Kumamoto, Kumamoto 860-0811 Japan; 3 Department of Molecular Imaging and Theranostics, National Institute of Radiological Sciences, National Institutes for Quantum and Radiological Science and Technology, 4-9-1 Anagawa, Inage-ku, Chiba, Chiba 263-8555 Japan

## Abstract

Nuclear medicine examinations for imaging gliomas have been introduced into clinical practice to evaluate the grade of malignancy and determine sampling locations for biopsies. However, these modalities have some limitations. Tissue factor (TF) is overexpressed in various types of cancers, including gliomas. We thus generated an anti-human TF monoclonal antibody (mAb) clone 1849. In the present study, immunohistochemistry performed on glioma specimens using anti-TF 1849 mAb showed that TF expression in gliomas increased in proportion to the grade of malignancy based on the World Health Organization (WHO) classification, and TF was remarkably expressed in necrosis and pseudopalisading cells, the histopathological hallmarks of glioblastoma multiforme (GBM). Furthermore, in both fluorescence and single-photon emission computed tomography/computed tomography (SPECT/CT) imaging studies, anti-TF 1849 IgG efficiently accumulated in TF-overexpressing intracranial tumours in mice. Although further investigation is required for a future clinical use of immuno-SPECT with ^111^In-labelled anti-TF 1849 IgG, the immuno-SPECT may represent a unique imaging modality that can visualize the biological characteristics of gliomas differently from those obtained using the existing imaging modalities and may be useful to evaluate the grade of malignancy and determine sampling locations for biopsies in patients with glioma, particularly GBM.

## Introduction

Gliomas are the most common type of malignant tumours originating in the central nervous system^1^. The treatment plan for gliomas is selected based on histopathological diagnosis. Each definitively diagnosed glioma is classified into four grades of malignancy according to the World Health Organization (WHO) classification, and the prognosis of patients with glioma depends on the grade of malignancy^2^. Although surgical resection is performed based on the policy of maximum safe resection as a first step in the treatment of patients with suspicion of glioma^3^ and the tumour samples resected surgically are used for histopathological diagnosis, tumour samples are obtained through biopsies in patients with tumours located at surgically inaccessible lesions or without tolerability of surgery under general anaesthesia. However, it has been reported that gliomas demonstrate significant intratumoural heterogeneity^4^, and the sampling error and small quantity of tumour samples obtained through biopsies can lead to inadequate histopathological diagnoses^5^. For example, the presence of necrosis typically surrounded by pseudopalisading cells and/or microvascular proliferation is essential for the histopathological diagnosis of glioblastoma multiforme (GBM), classified as the most malignant grade 4 glioma based on the WHO classification, and these histopathological hallmarks distinguish GBM from other gliomas. Therefore, if biopsy specimens do not contain necrosis and microvascular proliferation, then the histopathological examination could lead to a misdiagnosis, resulting in the underestimation of the grade. Indeed, a previous study in which a series of patients who underwent tumour resection following tumour biopsy were reviewed revealed that the discrepancy between histopathological diagnoses based on biopsy and tumour resection was 38%^5^.

In addition to conventional magnetic resonance imaging (MRI), several methods for imaging gliomas have been introduced into clinical practice to improve treatment results and diagnostic accuracy^6–9^. With regard to nuclear medicine imaging, there are modalities to evaluate the increased activity of membrane transporters expressed in tumours, such as glucose, amino acid and nucleoside transporters^6,7^. Among the modalities, [^11^C-methyl]-methionine (^11^C-MET) positron emission tomography (PET) is one of the most common modalities used in gliomas because of the generally low uptake in the normal brain, high uptake in the tumour and convenient synthesis of the tracer with high radiochemical purity^8,10^. Although ^11^C-MET PET is used for the medical care of gliomas to evaluate the grade of malignancy^8,9^, there are certain limitations to the accuracy of the evaluation because of overlap in ^11^C-MET uptake between each grade^8,9,11–14^. Additionally, ^11^C-MET PET is also used to determine the sampling location for biopsies^7–9,15,16^. However, previous studies comparing ^11^C-MET uptake at biopsy sites and the histopathological findings of tumour samples obtained using biopsies showed that ^11^C-MET uptake at sites with necrosis decreased compared with that at sites without necrosis, whereas positive correlations were observed between ^11^C-MET uptake and cell density and between ^11^C-MET uptake and cell proliferation^17–19^.

Patients with malignancies, including brain tumour, have a higher risk of venous thromboembolism compared with patients without malignancy^20^. This phenomenon suggests a systemic abnormality of the blood coagulation system in patients with malignancies. Moreover, in patients with GBM, intravascular thrombosis^21^ and fibrin deposition^22^ in surgically resected specimens are microscopically identified with high frequency. These histopathological findings indicate a blood coagulation system abnormality in the tumours of patients with GBM in addition to the systemic abnormalities. Tissue factor (TF), an initiator in the extrinsic pathway of coagulation, is a 47-kDa transmembrane glycoprotein that plays an important role in haemostasis^23^. In addition, TF is highly expressed in various types of cancers through mutations in proto-oncogenes and tumour suppressor genes or the hypoxic tumour microenvironment^24^. TF modulates pathological mechanisms in cancer such as cell proliferation, tumour invasion and metastasis^25^. Moreover, TF expression in tumour tissue is associated with poor prognosis in various types of cancers^24^. With regard to glioma, several studies have shown that TF expression is regulated through epidermal growth factor receptor (EGFR)^26^, epidermal growth factor receptor variant III (EGFRvIII) through the ligand-independent activation of EGFR^26,27^, phosphatase and tensin homolog deleted on chromosome 10 (PTEN)^28^ and hypoxia^28^, and TF is associated with the formation of necrosis surrounded by pseudopalisading cells and microvascular proliferation^29^. In addition, the expression level of TF in surgically resected glioma specimens is correlated with the WHO-classified malignancy grade^30^.

Recently, we generated an anti-human TF monoclonal antibody (mAb), namely clone 1849^31^. Furthermore, we reported that the antibody could be used as a probe for fluorescence imaging and showed the usefulness of the probe in a TF-overexpressing human pancreatic cancer xenograft model^32^.

In the present study, we investigated anti-TF 1849 mAb as an imaging probe for grading gliomas and determining sample locations for biopsies.

## Results

### Expression of TF in glioma surgical specimens evaluated by anti-TF 1849 mAb

**Figure 1:**
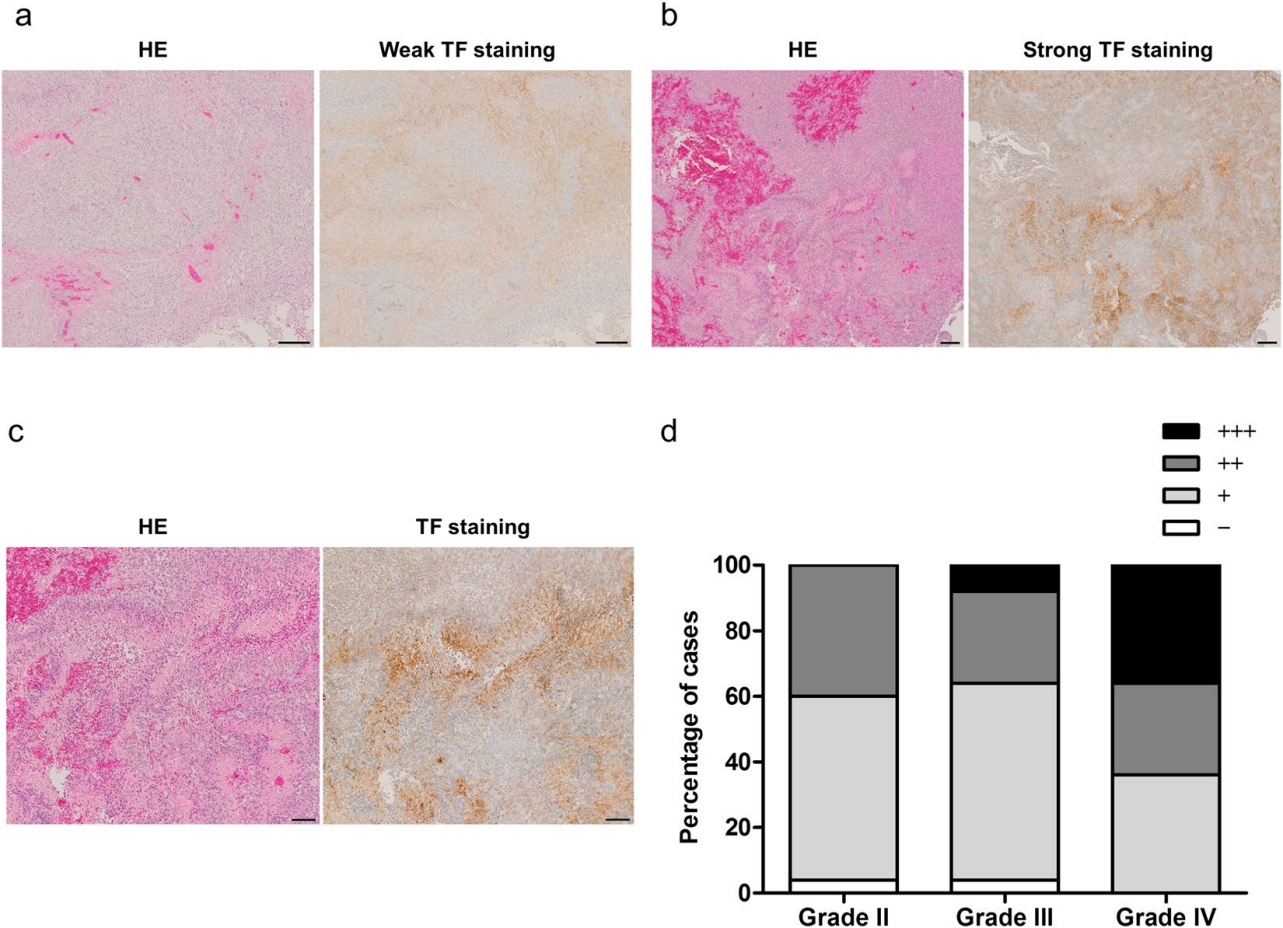
Immunohistochemical staining of TF expression in glioma specimens performed using anti-TF 1849 mAb. (**a**,**b**) The left panels show HE staining and the right panels show TF staining with weak intensity (**a**) and strong intensity (**b**) in GBM. Scale bar, 200 µm. (**c**) High-power field of (**b**). HE staining (left panel) shows morphological findings of necrosis surrounded by pseudopalisading cells in GBM. TF was highly expressed in necrosis and pseudopalisading cells (right panel). Scale bar, 100 µm. (**d**) The level of TF was classified into four degrees: (−), negative; (+), <50% positive tumour cells; (++), ≥50% positive tumour cells with weak intensity; and (+++), ≥50% positive tumour cells with strong intensity. The percentages of the four categories in grade 2, 3 and 4 gliomas are shown. Each group consisted of 25 cases.

To evaluate the expression of TF in gliomas, immunohistochemical staining of glioma specimens resected surgically was performed using anti-TF 1849 mAb. The expression of TF in glioma specimens was detectable using this antibody, and the staining intensity was determined as negative, weak and strong intensities (Fig. 1a and b). The staining was classified into four categories: (−), negative; (+), weakly positive (<50% positive tumour cells); (++), moderately positive (≥50% positive tumour cells with weak intensity); and (+++), strongly positive (≥50% positive tumour cells with strong intensity) as previously described^30^. Notably, TF was highly expressed in necrosis and pseudopalisading cells of GBMs (Fig. 1c). These pathological characteristics are representative of GBM. Collectively, the expression of TF in grade 4 gliomas was higher than that in grade 2 and grade 3 gliomas (Fig. 1d). In grade 4 gliomas, 36% of cases (9/25) were strongly positive, 28% of cases (7/25) were moderately positive, 36% of cases (9/25) were weakly positive, and 0% of cases (0/25) were negative. In grade 3 gliomas, 8% of cases (2/25) were strongly positive, 28% of cases (7/25) were moderately positive, 60% of cases (15/25) were weakly positive, and 4% of cases (1/25) were negative. In grade 2 gliomas, 0% of cases (0/25) were strongly positive, 40% of cases (10/25) were moderately positive, 56% of cases (14/25) were weakly positive, and 4% of cases (1/25) were negative (Fig. 1d).

### Expression of TF in normal cerebral lobes and cerebrum adjacent to tumour

**Figure 2:**
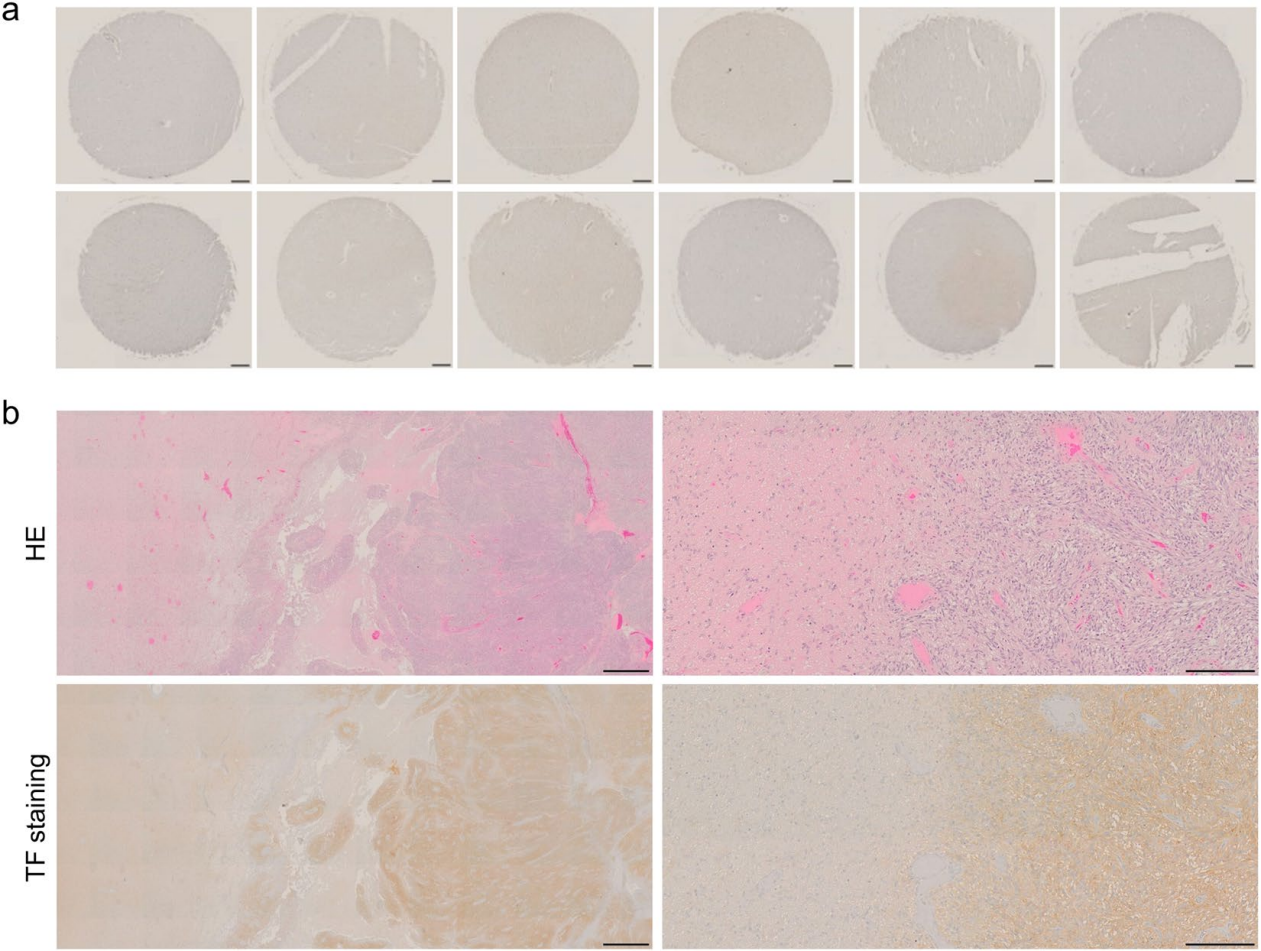
TF expression in normal cerebral lobes and cerebrum adjacent to tumour. (**a**) TF staining of a human normal brain tissue array. TF expression in the cerebral lobes was remarkably lower than that in the glioma surgical specimens. Scale bar, 200 µm. (**b**) HE staining (upper panels) and TF staining (lower panels) of a GBM specimen containing tumour and surrounding cerebrum. Compared with TF expression in the tumour (right side), the expression level in the surrounding cerebral white matter (left side) was lower (left panels). There was an obvious difference in the intensity of TF staining between the tumour (right side) and surrounding cerebral white matter (left side) at the invasive tumor front (right panels). Scale bar: left panels, 1 mm; right panels, 200 µm.

To evaluate TF expression in normal brain, immunohistochemistry with anti-TF 1849 mAb was performed on a human normal brain tissue array. The immunostaining revealed that TF expression in the cerebral lobes (Fig. 2a), from which gliomas usually arise, was remarkably lower than that in the glioma surgical specimens (Fig. 1a–c). Furthermore, immunohistochemical staining of a GBM specimen containing tumour and surrounding cerebrum was performed using anti-TF 1849 mAb (Fig. 2b). Compared with TF expression in the tumour (right side), the expression in the surrounding cerebral white matter (left side) was lower (Fig. 2b, left panels). At the invasive tumour front, there was an obvious difference in the intensity of TF staining between the tumour and surrounding cerebral white matter (Fig. 2b, right panels).

### Expression of TF in human glioma cell lines

**Figure 3:**
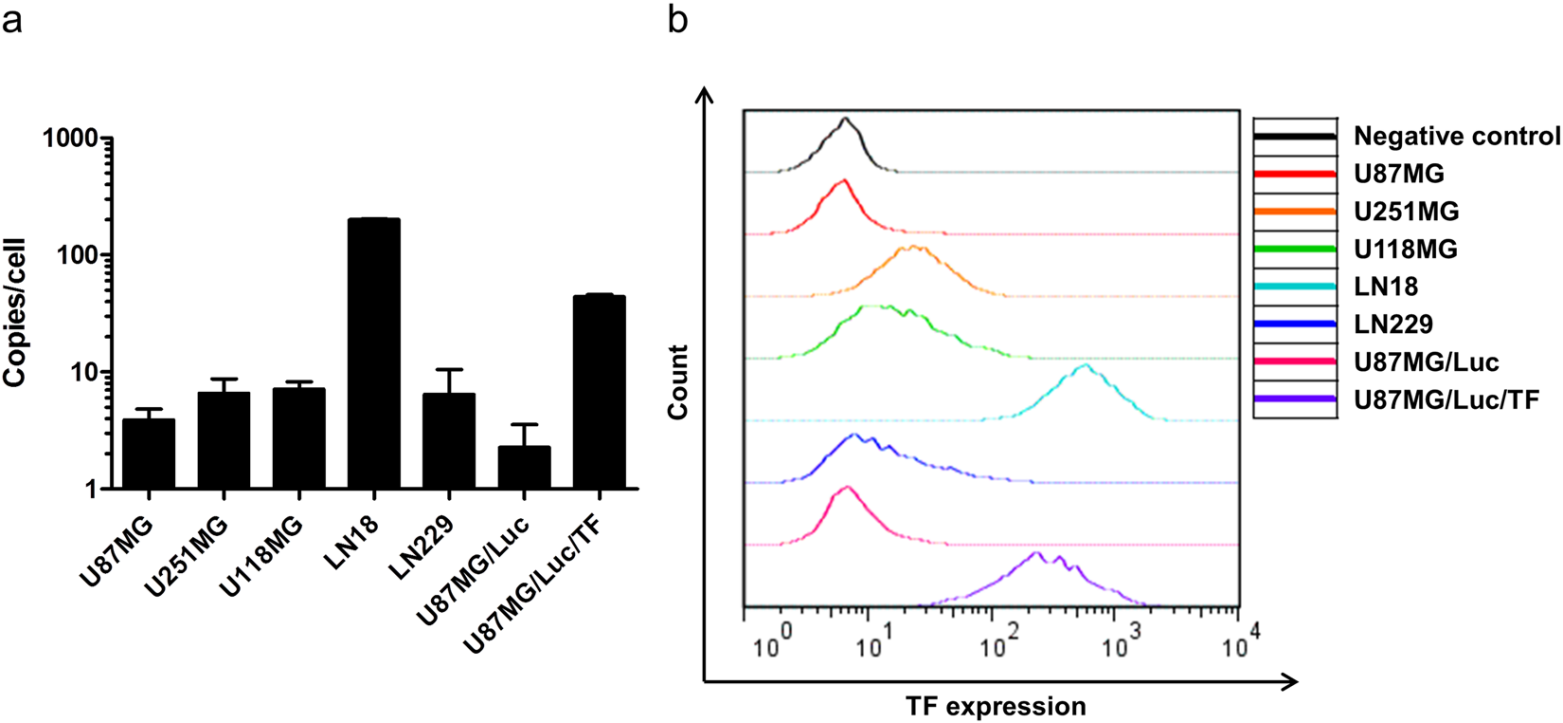
Expression of human TF in glioma cell lines. (**a**) Human TF copy number of glioma cell lines was determined using quantitative real-time RT-PCR. Human TF copy number in U87MG, U251MG, U118MG, LN18, LN229, U87MG/Luc and U87MG/Luc/TF was 3.90 ± 0.94 copies/cell, 6.57 ± 2.14 copies/cell, 7.16 ± 1.08 copies/cell, 200.36 ± 3.58 copies/cell, 6.44 ± 4.04 copies/cell, 2.77 ± 1.28 copies/cell and 44.13 ± 1.64 copies/cell, respectively. The experiments were repeated three times, and the data are shown as the means ± standard deviation (SD). (**b**) Expression of human TF protein on glioma cell lines was evaluated using flow cytometry. Relative expression of human TF protein normalized to the negative control in U87MG, U251MG, U118MG, LN18, LN229, U87MG/Luc and U87MG/Luc/TF was 0.98, 3.73, 2.44, 85.96, 1.82, 1.16 and 40.83, respectively.

The human TF copy number determined using real-time reverse transcription-polymerase chain reaction (RT-PCR) in U87MG, U251MG, U118MG, LN18 and LN229 was 3.90 ± 0.94 copies/cell, 6.57 ± 2.14 copies/cell, 7.16 ± 1.08 copies/cell, 200.36 ± 3.58 copies/cell and 6.44 ± 4.04 copies/cell, respectively (Fig. 3a). Expression of human TF protein in these cell lines was evaluated using flow cytometry. The relative expression of human TF protein normalized to the negative control in U87MG, U251MG, U118MG, LN18 and LN229 was 0.98, 3.73, 2.44, 85.96 and 1.82, respectively (Fig. 3b). The results of real-time RT-PCR and flow cytometry analyses showed that human TF expression was the highest in the LN18 cell line. Unfortunately, the LN18 cell line appeared to be non-tumourigenic in nude mice. Therefore, we transduced the human TF gene into the U87MG/Luc cell line using a lentiviral vector and established a TF-overexpressing cell line with stable tumourigenicity (U87MG/Luc/TF). The human TF copy number in U87MG/Luc and U87MG/Luc/TF was 2.77 ± 1.28 copies/cell and 44.13 ± 1.64 copies/cell, respectively (Fig. 3a). The relative expression of human TF protein normalized to the negative control in U87MG/Luc and U87MG/Luc/TF was 1.16 and 40.83, respectively (Fig. 3b).

### The affinity and specificity of fluorescent- and radiolabelled antibodies for TF expression in glioma cell lines

**Figure 4:**
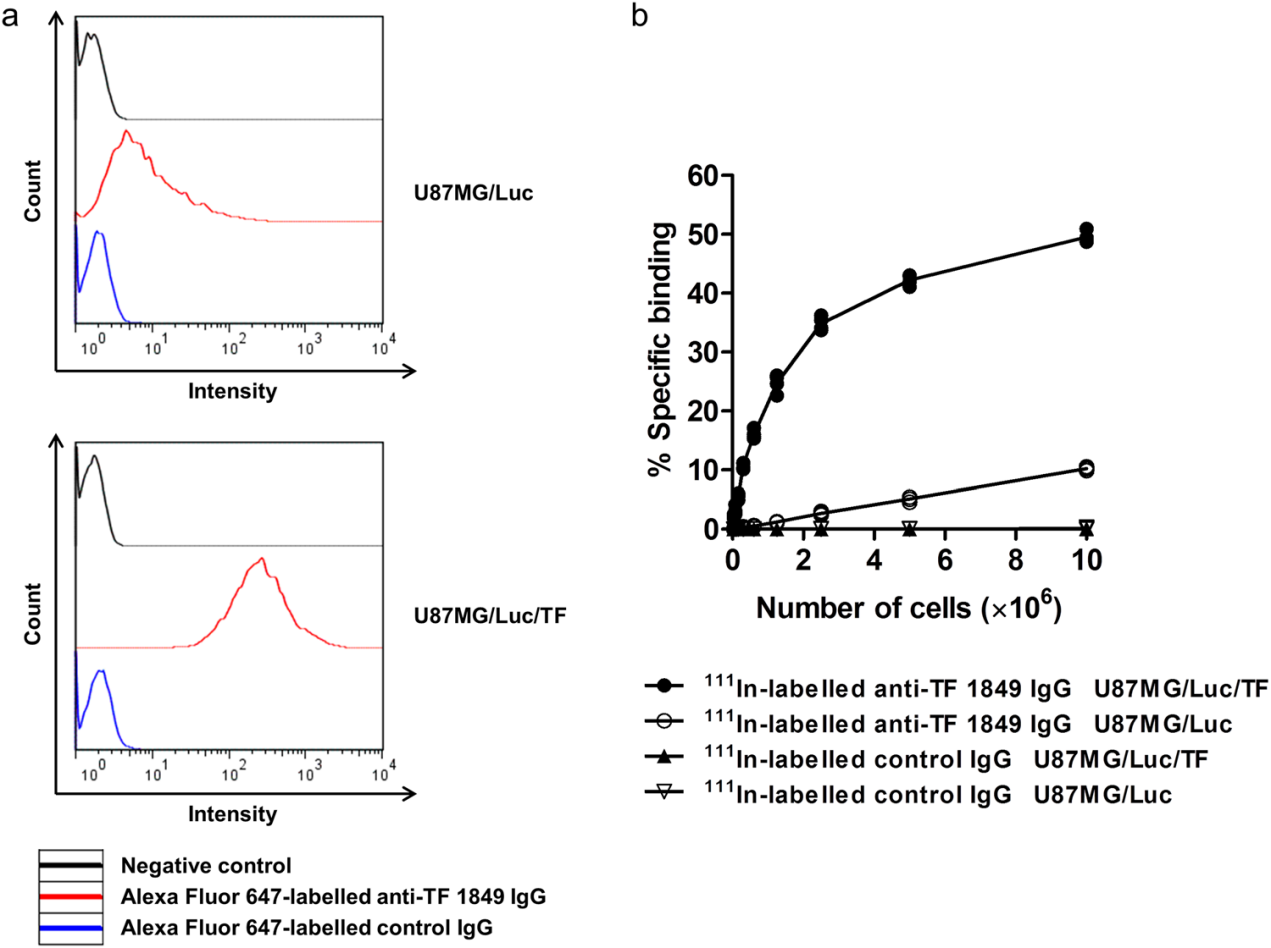
The affinity and specificity of fluorescent- and radiolabelled antibodies for TF expression in glioma cell lines. (**a**) The affinity of Alexa Fluor 647-labelled anti-TF 1849 IgG and control IgG for TF expressed on U87MG/Luc (upper) and U87MG/Luc/TF cells (lower) was evaluated using flow cytometry. The relative intensity of Alexa Fluor 647-labelled anti-TF 1849 IgG normalized to the negative control in U87MG/Luc and U87MG/Luc/TF was 4.25 and 158.06, respectively. The relative intensity of Alexa Fluor 647-labelled control IgG in U87MG/Luc and U87MG/Luc/TF was 1.23 and 1.28, respectively. (**b**) To evaluate the affinity and specificity of ^111^In-labelled anti-TF 1849 IgG and control IgG, we examined the capacity of the radiolabelled antibodies to bind to serially diluted U87MG/Luc and U87MG/Luc/TF cells. The radioactivity derived from ^111^In-labelled anti-TF 1849 IgG bound to U87MG/Luc/TF cells increased in a cell number-dependent manner. In contrast, the increased radioactivity in U87MG/Luc cells was considerably lower compared to in U87MG/Luc/TF cells. However, ^111^In-labelled control IgG showed negligible binding to both U87MG/Luc and U87MG/Luc/TF cells.

The affinity and specificity of Alexa Fluor 647-labelled antibodies were evaluated using flow cytometry. The data showed that Alexa Fluor 647-labelled anti-TF 1849 IgG showed low affinity against U87MG/Luc cells and high affinity against U87MG/Luc/TF cells. The relative intensity of the fluorescent anti-TF 1849 IgG normalized to the negative control in U87MG/Luc and U87MG/Luc/TF was 4.25 and 158.06, respectively (Fig. 4a). However, Alexa Fluor 647-labelled control IgG had negligible affinity against both U87MG/Luc and U87MG/Luc/TF cells. The relative intensity of the fluorescent control IgG normalized to the negative control in U87MG/Luc and U87MG/Luc/TF was 1.23 and 1.28, respectively (Fig. 4a).

With regard to ^111^In-labelled antibodies in the present study, cellulose acetate electrophoresis revealed a conjugation ratio of DTPA to antibodies of 1.1, and the radiochemical yields were 72% to 90%. The radiochemical purity was more than 97%, as determined through thin-layer chromatography using 70% methanol as the mobile phase. The specific activities were 22 to 54 kBq/µg. In the cell binding assay, ^111^In-labelled anti-TF 1849 IgG highly bound to U87MG/Luc/TF cells and the radioactivity derived from the antibody bound to U87MG/Luc/TF cells increased in a cell number-dependent manner. In contrast, the increase in the radioactivity in U87MG/Luc cells was considerably lower compared with that in U87MG/Luc/TF cells (Fig. 4b). However, ^111^In-labelled control IgG showed negligible binding to both U87MG/Luc and U87MG/Luc/TF cells (Fig. 4b).

### *Ex vivo* fluorescence imaging

**Figure 5:**
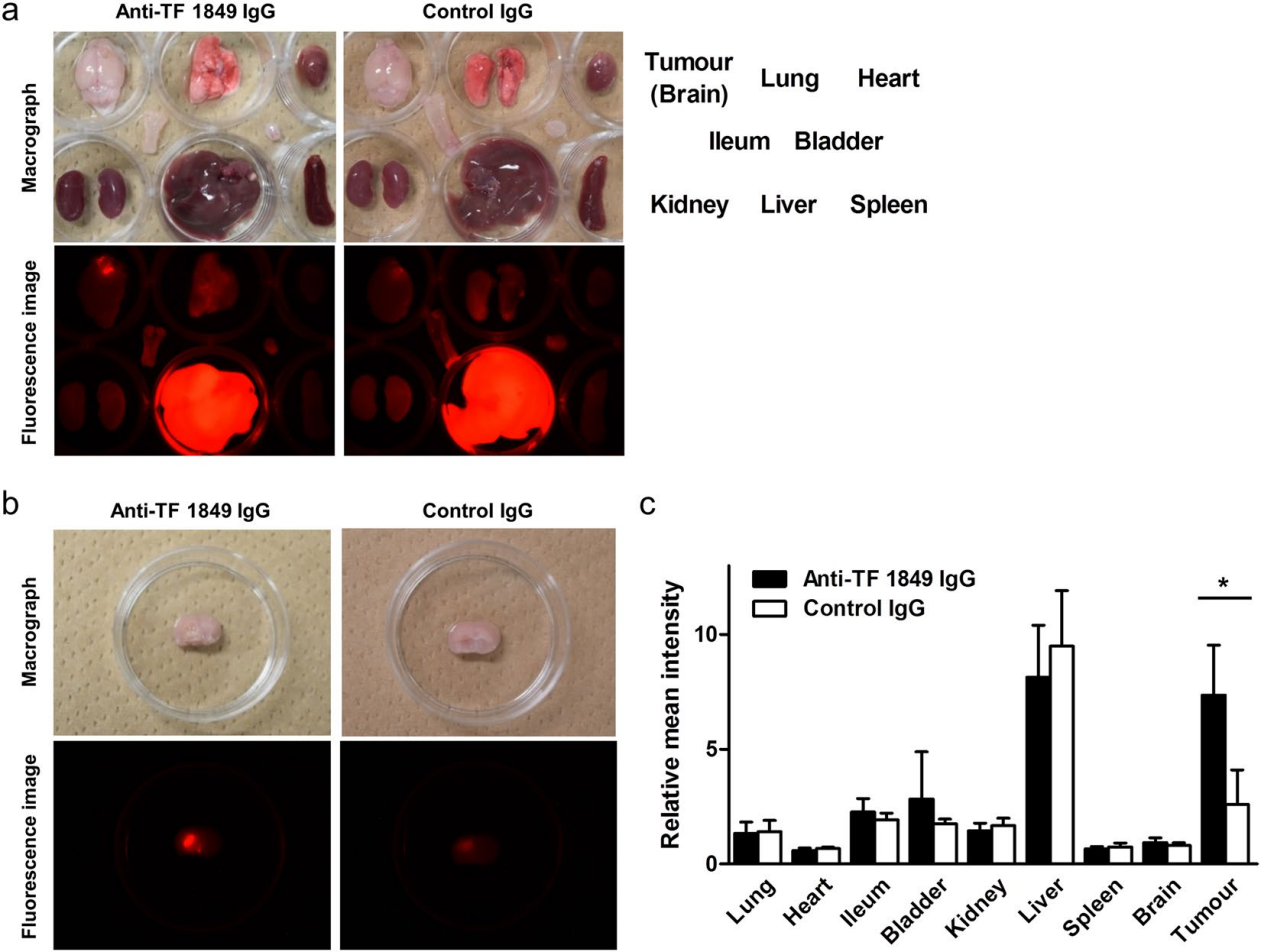
*Ex vivo* fluorescence imaging study. (**a**,**b**) The findings of the *ex vivo* fluorescence imaging study at 1 day after the injection. The upper and lower panels show macrographs and fluorescence images, respectively. Alexa Fluor 647-labelled anti-TF 1849 IgG (left panels) more efficiently accumulated in the intracranial tumours overexpressing TF compared with Alexa Fluor 647-labelled control IgG (right panels). (**c**) Quantitative analysis of the *ex vivo* fluorescence imaging study showed significant differences in the relative mean intensity of the intracranial tumours between the fluorescent anti-TF 1849 IgG-administered and control IgG-administered groups. However, there was no significant difference in the other organs between both groups. *N* = 4 mice per group. The data are shown as the means ± SD. *P = 0.029.

*Ex vivo* fluorescence imaging revealed that Alexa Fluor 647-labelled anti-TF 1849 IgG more efficiently accumulated in the intracranial tumour overexpressing TF compared with Alexa Fluor 647-labelled control IgG (Fig. 5a and b). Quantitative evaluation revealed significant differences in the relative mean intensity of the intracranial tumours between the fluorescent anti-TF 1849 IgG-administered and control IgG-administered groups (*P* = 0.029) (Fig. 5c). In contrast, there was no significant difference in the other organs between both groups (Fig. 5c).

### Distribution of fluorescent antibodies in tumour tissues

**Figure 6:**
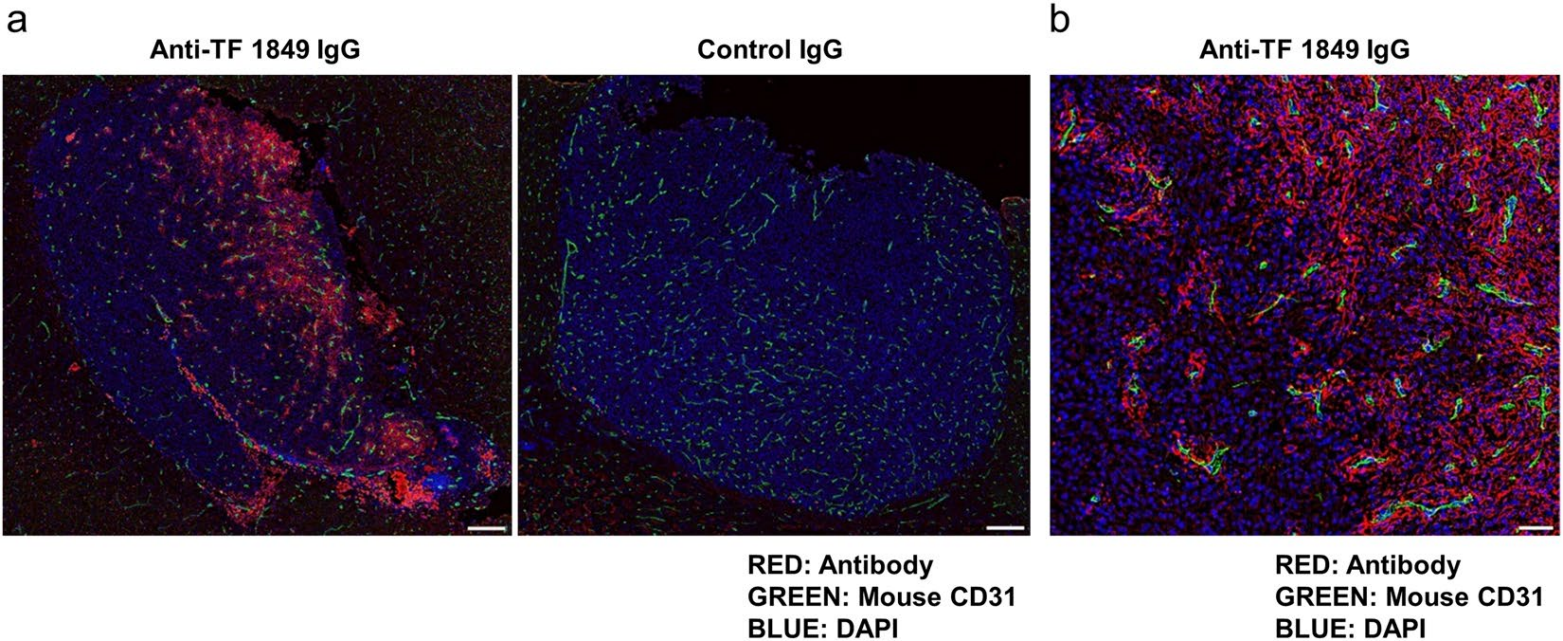
Distribution of fluorescent antibodies in the intracranial tumour tissues. (**a**) The panels show the intracranial tumour tissues in the Alexa Fluor 647-labelled anti-TF 1849 IgG-administered mice (left panel) and Alexa Fluor 647-labelled control IgG-administered mice (right panel). In the fluorescent anti-TF 1849 IgG-administered group, the antibodies were observed in the intracranial tumour tissues. In contrast, in the fluorescent control IgG-administered group, the antibodies in the tumour tissues were hardly detectable. The Alexa Fluor 647-labelled antibodies, endothelial cells, and nucleus are indicated in red, green and blue, respectively. Scale bar, 200 µm. (**b**) High-power field of the tumour section in the Alexa Fluor 647-labelled anti-TF 1849 IgG-administered mice. Anti-TF 1849 IgGs were located on cell membranes of TF-overexpressing tumour cells around blood vessels. Scale bar, 50 µm.

We subsequently microscopically evaluated the distribution of the Alexa Fluor 647-labelled antibodies in the intracranial tumour tissues. In the fluorescent anti-TF 1849 IgG-administered group, fluorescence signals were detected in the intracranial tumour tissues (Fig. 6a). In contrast, fluorescence signals in the tumour tissues were negligible in the fluorescent control IgG-administered group (Fig. 6a). Although the intratumoural accumulation of control IgG was observed in *ex vivo* fluorescence imaging study, the fluorescence signals of control IgGs in the tumour tissues were negligible. Magnifying observation of the tumour tissues in the fluorescent anti-TF 1849 IgG-administered group revealed that anti-TF 1849 IgGs were located on the cell membranes of the TF-overexpressing tumour cells surrounding the blood vessels (Fig. 6b).

### SPECT/CT

**Figure 7:**
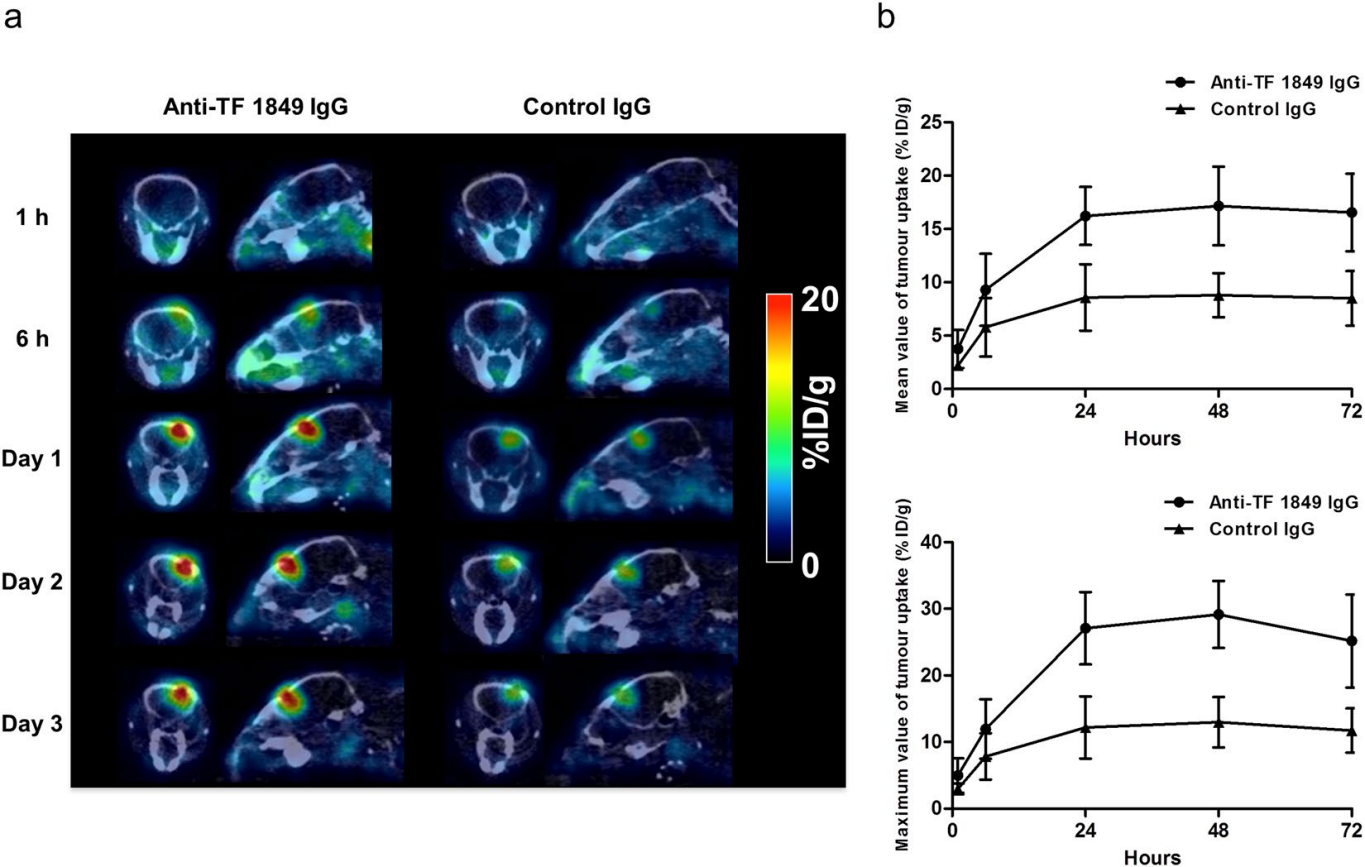
SPECT/CT study. (**a**) Representative SPECT/CT images. ^111^In-labelled anti-TF 1849 IgG (left) more efficiently accumulated in the intracranial tumours overexpressing TF compared with ^111^In-labelled control IgG (right). (**b**) Quantitative analysis of the SPECT/CT study revealed significant differences in the mean (upper) and maximum (lower) values of tumour uptake between the ^111^In-labelled anti-TF 1849 IgG-administered and control IgG-administered groups (*P* < 0.01). *N* = 4 mice per group. The data are shown as the means ± SD. Points, mean; bars, SD.

To visualize the accumulation of the antibodies in the intracranial tumour *in vivo*, we conducted single-photon emission computed tomography/computed tomography (SPECT/CT) imaging using ^111^In-labelled antibodies. Fused SPECT/CT images showed that ^111^In-labelled anti-TF 1849 IgG remarkably accumulated in intracranial tumours overexpressing TF compared with ^111^In-labelled control IgG (Fig. 7a). Quantitative evaluation revealed significant differences in the mean and maximum values of tumour uptake between the ^111^In-labelled anti-TF 1849 IgG-administered group and the control IgG-administered group (*P* < 0.01), and the accumulation of anti-TF 1849 IgG in the intracranial tumour was approximately twice as efficient as that of control IgG at 24 h after injection (Fig. 7b).

### Autoradiography

**Figure 8:**
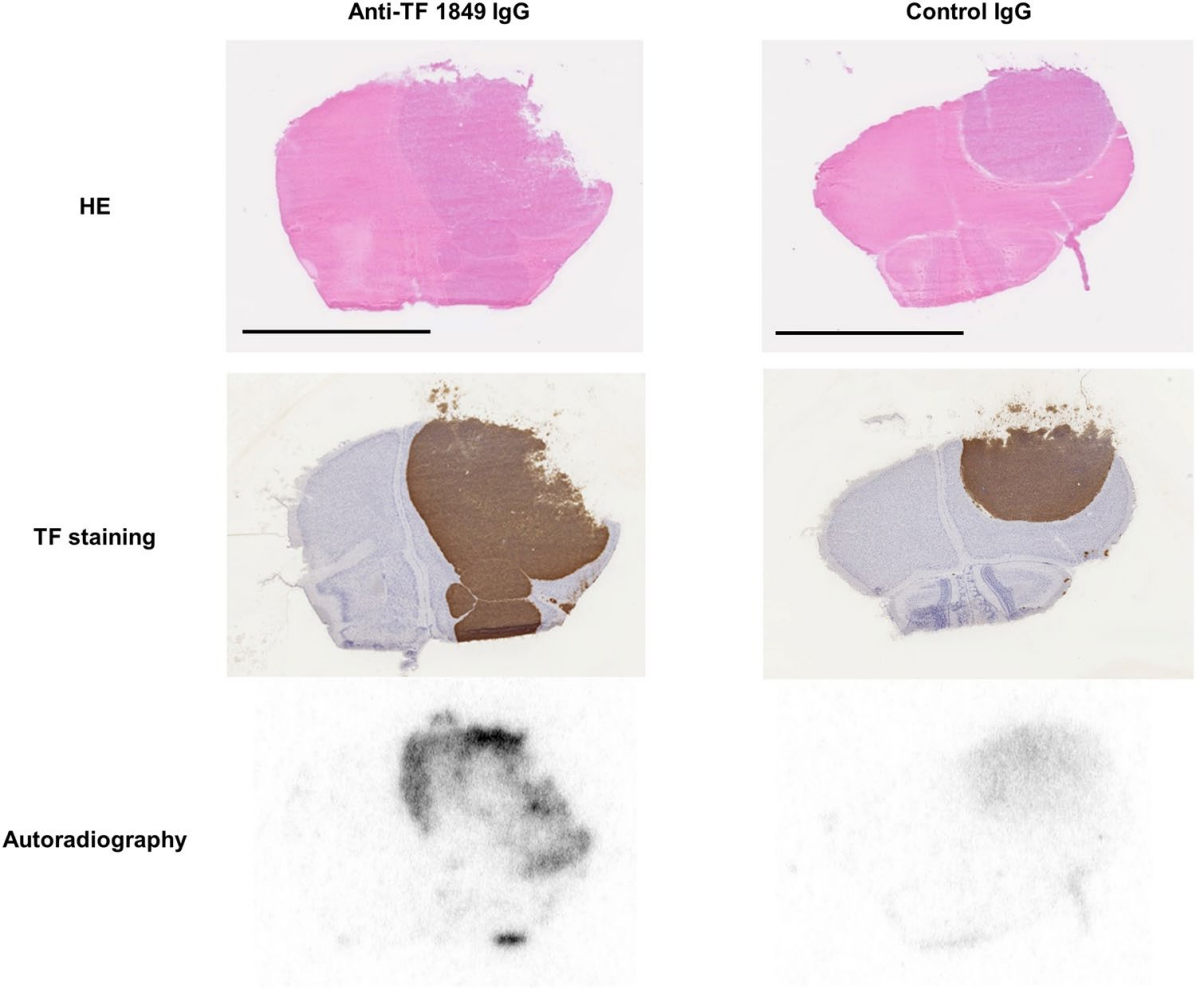
Autoradiography. The upper, middle and lower panels show HE staining, TF immunostaining and autoradiography, respectively. The radioactivity derived from ^111^In-labelled antibodies was observed in the intracranial tumour tissues in both the anti-TF 1849 IgG-administered (left panels) and control IgG-administered mice (right panels), whereas the radioactivity in normal brain tissues was barely detectable in both groups. Autoradiography confirmed that ^111^In-labelled anti-TF 1849 IgG (lower left panel) more efficiently accumulated in the intracranial tumours overexpressing TF compared with ^111^In-labelled control IgG (lower right panel). Anti-TF 1849 IgGs were heterogeneously distributed in the tumour tissues (lower left panel), whereas TF immunostaining showed that TF was homogeneously expressed in the tumours (middle left panel). Scale bar, 4 mm.

Autoradiography showed that the radioactivity derived from ^111^In-labelled antibodies was observed in the intracranial tumour tissues in both the ^111^In-labelled anti-TF 1849 IgG-administered and control IgG-administered groups, whereas radioactivity was barely detectable in normal brain tissues (Fig. 8). These autoradiography findings were consistent with fused SPECT/CT images showing high contrast between intracranial tumour and normal brain in both groups. Moreover, autoradiography confirmed the increased efficient accumulation of anti-TF 1849 IgG in intracranial tumours overexpressing TF compared with control IgG (Fig. 8). Anti-TF 1849 IgGs were heterogeneously distributed in the tumour tissues, whereas immunohistochemical staining of the intracranial tumours showed that TF was homogeneously expressed in the tumours (Fig. 8). The heterogeneous distribution indicated that the penetration of anti-TF 1849 IgG into the intracranial tumours was partially restricted.

## Discussion

In the present study, we evaluated the potential clinical application of anti-human TF mAb (clone 1849) for molecular imaging of gliomas.

First, we performed immunohistochemistry for surgically resected WHO-classified glioma specimens using an anti-TF 1849 mAb. The immunohistochemical examination revealed higher TF expression in grade 4 gliomas than in lower-grade gliomas. TF expression was particularly strong in the area of necrosis and pseudopalisading cells of GBM. These findings were consistent with those of previous studies^28,30^. The results in the present immunohistochemical study suggested that TF expression in gliomas was useful for evaluating the malignancy grade, and remarkable TF expression in gliomas represented the presence of necrosis and pseudopalisading cells, which are pivotal characteristics of GBM. In addition, the present study revealed that clone 1849 had a sufficient affinity for human TF expressed in glioma specimens.

Second, we evaluated the tumour accumulation of anti-TF 1849 IgG in model mice with intracranial tumours overexpressing TF. *Ex vivo* fluorescence imaging showed that the fluorescence intensity in the intracranial tumour was stronger in the anti-TF 1849 IgG-administered group than in the control IgG-administered group, and that in the normal brain was negligible in both groups. Fused SPECT/CT images showed higher contrast between the tumour and normal brain in the anti-TF 1849 IgG-administered group compared with the control IgG-administered group. The findings of fused SPECT/CT images were confirmed through autoradiography in the same specimens. The findings of *ex vivo* fluorescence and SPECT/CT imaging studies suggest that the blood brain barrier (BBB) in tumours is partially broken and that IgG, the molecular weight of which is approximately 150 kDa, leaks from brain vessels into tumour tissue. After leaking from brain vessels, even non-targeting IgG antibodies accumulate in tumour tissue based on the enhanced permeability and retention (EPR) effect^33^. Previous studies have reported that glioma cells transplanted into the rodent brain migrate along the brain vasculature, proliferate *en route* and eventually encase the vessels^34^. The transplanted cells intercalate between endothelial cells and perivascular astrocytes and interrupt the interaction between the cells, resulting in the breakdown of the BBB and increased vascular permeability^35,36^. In addition to the observations in clinically relevant models of glioma, several studies have reported morphological alterations in endothelial cells, pericytes and the perivascular space, and the down-regulation and redistribution of proteins contribute to the formation of the BBB in gliomas^37–39^. The findings of imaging examinations in patients with GBM, such as the elevated relative cerebral blood volume and extravasation of contrast agent in tumours, also indicate the increase in vascularity and breakdown of the BBB as characteristics of GBM^6^. Moreover, in preclinical studies, we previously reported that macromolecules such as polymeric micelle incorporating anti-cancer drugs accumulated in intracranial tumours of glioma model mice based on the EPR effect^40,41^. In the present study, *ex vivo* fluorescence and SPECT/CT imaging studies also revealed that anti-TF 1849 IgG more efficiently accumulated in intracranial tumours overexpressing TF compared with control IgG. Thus, we proposed that in addition to the EPR effect, active targeting based on antigen-antibody reactions enhances the accumulation of antibodies in intracranial tumours with a partially disrupted BBB. In contrast to the accumulation of the antibodies in the intracranial tumour, that in the normal brain was remarkably lower in the present study. It is known that an intact BBB is probably responsible for the low accumulation^42^. However, since clone 1849 does not recognize mouse TF antigen, we cannot estimate the effect of anti-TF 1849 IgG on normal brain tissue in clinics. However, the uptake in brain adjacent to tumour may be minor, because, in addition to the theory that the normal BBB prevents antibodies entering brain^42^, TF staining in the cerebral lobes of the normal brain tissue array and cerebral white matter adjacent to the tumour was remarkably lower than that in the tumour.

The autoradiographic and immunohistochemical studies revealed that anti-TF 1849 IgGs were heterogeneously distributed in the tumour tissues although TF was homogeneously overexpressed in the tumours. In addition, the microscopic observation of the tumour tissues in the fluorescent anti-TF 1849 IgG-administered mice showed that anti-TF 1849 IgGs were heterogeneously distributed at the region close to blood vessels. These findings suggest that heterogeneous vascular permeability and the binding site barrier effect^43–45^ are responsible for the heterogeneous distribution of anti-TF 1849 IgGs in the tumours. Moreover, it is assumed that elevated interstitial pressure in intracranial tumours also affect the penetration of antibodies within tumours^46,47^.

We also demonstrated that anti-TF 1849 IgG could be used as a probe for SPECT analysis through antibody labelling with ^111^In for the successful visualization of TF expression in intracranial tumours *in vivo*.

Previous studies have demonstrated successful visualization of TF expressed in pancreatic or breast cancer models by PET imaging with a full-length anti-human TF antibody^48^, its antigen-binding fragment (Fab)^49^, a bispecific heterodimer composed of anti-human TF and anti-human/murine CD105 Fab fragments^50,51^ and an active site-inhibited factor VIIa^52,53^, and proposed that PET imaging of TF can be used for assessing disease stage and progression in patients with malignancy and used as a companion diagnostics for TF-targeted therapies. In the present study, we succeeded in imaging TF expression in tumours of the orthotopic glioma model mice using our original mAb. Moreover, based on the results of our immunohistochemical study, we may be able to use SPECT imaging of TF to evaluate malignancy grade and determine sampling locations for biopsies in patients with glioma. Since we can produce radionuclides for SPECT without cyclotron, the radionuclides for SPECT can be obtained more easily compared with those for PET^54^. Therefore, in the present study, we decided to select the radionuclide for SPECT to visualize TF expression in tumours and it is meaningful that anti-TF 1849 IgG can be used as a probe for SPECT with higher convenience. However, the spatial resolution of PET is better than that of SPECT^54^. Therefore, in view of the imaging resolution, we need to examine whether the anti-TF 1849 mAb can also be used for a PET probe before proceeding to clinical studies.

In conclusion, the expression of TF in gliomas increased in proportion to the WHO-classified grade of gliomas. In particular, TF is highly expressed in necrosis and pseudopalisading cells, which are characteristics of GBM. Anti-TF 1849 IgG efficiently accumulated in intracranial tumours with high TF expression based on both active and passive targeting. Immuno-SPECT using ^111^In-labelled anti-TF 1849 IgG may become a unique imaging modality for the visualization of the biological characteristics of gliomas that is different from those obtained using the existing imaging modalities and may be useful to evaluate malignancy grade and determine sampling locations for biopsies in patients with glioma, particularly GBM. However, since various factors such as tumour vascular permeability, the binding site barrier effect and elevated interstitial pressure in intracranial tumours may affect the distribution of anti-TF 1849 IgG in tumour tissues, it should be required to investigate correlations between clone 1849 uptake in tumours and the malignancy grade or histopathological features such as necrosis and pseudopalisading cells. Finally, we are considering that clone 1849 should be humanized for a future clinical development of the immuno-SPECT.

## Methods

### Antibodies

Rat mAb IgG2b showing a high affinity for human TF antigen but not for mouse TF antigen (clone 1849) was established in the laboratory^31^. Rat mAb IgG2b recognizing neither human TF antigen nor mouse TF antigen (clone 372) was used as an isotype control antibody in these imaging studies.

### Immunohistochemistry of glioma and normal brain specimens

Glioma samples were obtained from the Department of Neurosurgery at Kumamoto University. Informed consent was obtained in writing and verbally from all patients, and the study was performed in accordance with the ethical guidelines of the 1975 Declaration of Helsinki and approved by the institutional review board of the Kumamoto University. A human normal brain tissue array was purchased commercially (US Biomax, Rockville, MD, USA). Tissue sections from paraffin-embedded samples were deparaffinised and incubated with 0.3% hydrogen peroxidase for 20 min at room temperature (RT) to block endogenous peroxidase. Heat-induced antigen retrieval was performed using citrate buffer adjusted to pH 6 (Dako, Glostrup, Denmark). After blocking with 5% skim milk in phosphate buffered saline (PBS), the slides were incubated with anti-TF 1849 mAb overnight at 4 °C. After washing with PBS, the slides were incubated with polymerized peroxidase-labelled goat anti-rat Fab’ (MBL Co., Ltd., Nagoya, Japan) for 1 h at RT. The reaction was visualized using 3,3′-diaminobenzidine (DAB; Dako), and hematoxylin was used for counter staining.

### Human glioma cell lines

The human glioma cell lines U87MG, U251MG, U118MG, LN18 and LN229 were obtained from the American Type Culture Collection (ATCC; Manassas, VA, USA). The cells were maintained in Dulbecco’s modified Eagle’s medium (Wako, Osaka, Japan) supplemented with 10% foetal bovine serum (Gibco, Grand Island, NY, USA), 100 units/ml penicillin, 100 µg/ml streptomycin and 0.25 µg/ml amphotericin B (Wako) at 37 °C in a humidified atmosphere containing 5% CO_2_.

### Establishment of U87MG expressing firefly luciferase

We established a U87MG cell line stably expressing firefly luciferase (U87MG/Luc) using Cignal Lenti Positive Control (Qiagen, Valencia, CA, USA) and SureENTRY Transduction Reagent (Qiagen) according to the manufacturer’s instructions.

### Establishment of U87MG/Luc forcibly expressing TF

U87MG/Luc cells were transduced with Precision LentiORF Collection (pLOC) vector containing human TF (Thermo Fisher Scientific, Wilmington, DE, USA) in accordance with the manufacturer’s instructions. After cell sorting conducted with a BD FACS Aria cell sorter (BD Biosciences, San Jose, CA, USA), the cells were cloned using a limiting dilution method.

### TF copy number in human glioma cell lines

Human TF copy number of glioma cell lines was determined using quantitative real-time RT-PCR as previously described^55^.

### Expression of TF protein in human glioma cell lines

Expression of human TF protein in glioma cell lines was determined through flow cytometry. The cells were harvested with non-enzymatic cell dissociation solution (Sigma-Aldrich, St. Louis, MO, USA) and suspended in PBS containing 0.1% bovine serum albumin and 2 mM EDTA (B. E. PBS). Aliquots of the cells (2 $\times$ 10^5^ cells) were dispensed into a 2-ml tube and incubated with 0.5 µg of anti-TF 1849 mAb for 30 min on ice. After washing with B. E. PBS, the cells were incubated with 1 µg of Alexa Fluor 647-conjugated anti-rat IgG (Life Technologies, Eugene, OR, USA) for 30 min on ice. Subsequently, the cells were washed with B. E. PBS and the nuclei were stained with propidium iodide (Invitrogen, Eugene, OR, USA). The samples were run on a Guava easyCyte (Millipore, Billerica, MA, USA), and the acquired data were analysed using FlowJo 7.5.5 software (Tree Star Inc., Ashland, OR, USA).

### Orthotopic glioma model

Six-week-old female BALB/c nu/nu mice (Charles River Japan, Yokohama, Japan) were used in the present study. We inoculated U87MG/Luc/TF cells (1 × 10^5^ cells) suspended in 3 µl of PBS into the right cerebral hemisphere of mice as previously described^40^. The study was approved by the Committees for Animal Experimentation of the National Cancer Center and National Institute of Radiological Sciences, Japan. All animal procedures were performed in compliance with the Guidelines for the Care and Use of Experimental Animals established by the Committees. These guidelines meet the ethical standards required by law and also comply with the guidelines for the use of experimental animals in Japan.

### Fluorescent labelling of antibodies

According to the manufacturer’s instructions, we prepared Alexa Fluor 647-labelled anti-TF 1849 IgG and control IgG using a protein labelling kit (Life Technologies).

### Radiolabelling of antibodies

^111^In-labelled anti-TF 1849 IgG and control IgG were prepared as previously described^56^. Briefly, the antibodies were conjugated with *p*-SCN-Bz-CHX-A”-DTPA (DTPA; Macrocyclics, Dallas, TX, USA), and DPTA-conjugated antibodies were purified with a Sephadex G-50 column (GE Healthcare, Little Chalfont, UK). Twenty micrograms of the conjugates was mixed with [^111^In]Cl_3_ (Nihon Medi-Physics, Tokyo, Japan) in 0.5 M acetate buffer (pH 6.0) and incubated for 30 min at RT. Subsequently, the radiolabelled antibodies were purified using a Sephadex G-50 column (GE Healthcare).

### The affinity and specificity of fluorescent- and radiolabelled antibodies for TF expression in glioma cell lines

We evaluated the affinity and specificity of Alexa Fluor 647-labelled anti-TF 1849 IgG and control IgG for TF expression in glioma cell lines through flow cytometry as described above using each fluorescent antibody as a primary antibody.

The affinity and specificity of ^111^In-labelled anti-TF 1849 IgG and control IgG for TF expression in glioma cell lines were evaluated as previously described^57^. In brief, serially-diluted U87MG/Luc and U87MG/Luc/TF cells in PBS containing 1% BSA were incubated with each radiolabelled antibody for 60 min on ice. After washing, the radioactivity bound to the cells was counted. The percentage of specific binding was calculated by dividing the radioactivity bound to the cells by the total radioactivity initially added.

### *Ex vivo* fluorescence imaging

The orthotopic glioma model mice were anaesthetized with isoflurane and intraperitoneally administered with D-luciferin potassium salt (Promega, Madison, WI, USA) at a dose of 125 mg/kg body weight, and bioluminescence images were acquired at 10 min after administration using the IVIS Kinetic imaging system (Caliper Life Sciences, Hopkinton, MA, USA). Regions of interest (ROIs) were drawn in the whole mouse head, and maximum radiances in ROIs were determined using IVIS Living Imaging 3.0 software (Caliper Life Sciences). The model mice with over 1 × 10^8^ p/s/cm/sr in the maximum radiance were enrolled in fluorescence imaging study and injected with Alexa Fluor 647-labelled anti-TF 1849 IgG or control IgG via the tail vein at a dose of 100 µg/mouse. The molar ratio of antibody and Alexa Fluor 647 in the fluorescent antibodies was adjusted by adding unlabelled antibody.

At 1 day after the injection, the mice were euthanized and the intracranial tumours with normal brain and major organs were excised and washed with isotonic sodium chloride solution, and *ex vivo* fluorescence images were acquired using the OV110 *in vivo* imaging system (Olympus, Tokyo, Japan). To exclude the effects that the depth of each tumour generated in the brain had on the quantification of the fluorescence intensity in the tumour, we coronally cut the brain tumour at the injection point and measured the mean intensity of the intracranial tumour on the coronal plane (Fig. 5b). With regard to the other organs including normal brain, we measured the mean intensities on the surface of each organ. The mean intensity of normal brain was determined on the left cerebral hemisphere. ROIs were manually drawn on the tumour and other organs, and the mean intensities were quantified with Image J 1.48 v in the same manner described previously^58^. The relative mean intensities were calculated by dividing the mean intensities of the tumour and organs by that of the normal cerebellum in each mouse.

### Distribution of fluorescent antibodies in tumour tissues

After the *ex vivo* fluorescence imaging study, each brain tumour was frozen in Tissue-Tec optimal-cutting-temperature compound (Sakura Finetek, Tokyo, Japan). Frozen sections (10 µm thick) were fixed with 4% paraformaldehyde in PBS for 15 min at RT and blocked with 5% skim milk in PBS overnight at 4 °C. Subsequently, the sections were incubated with 2 µg/ml goat anti-mouse CD31 polyclonal antibody (R&D Systems, Minneapolis, MN, USA) for 1 h at RT. After washing with PBS, the sections were incubated with 4 µg/ml Alexa Fluor 555-conjugated donkey anti-goat IgG polyclonal antibody (Invitrogen) for 1 h at RT. Subsequently, the nucleus was stained with DAPI for 5 min at RT. Fluorescence images were acquired using the VS120 fluorescence microscopy system (Olympus).

### SPECT/CT

The model mice enrolled in the SPECT/CT imaging study were determined as described in the fluorescence imaging study and injected with approximately 1.85 MBq of ^111^In-labelled anti-TF 1849 IgG or control IgG via the tail vein. The injected antibody dose was adjusted to 50 µg per mouse by adding the corresponding unlabelled antibody. At 1 and 6 h and 1, 2 and 3 days after the injection, the mice were anaesthetized with isoflurane and imaged with the VECTor/CT SPECT/CT Pre-Clinical Imaging system equipped with a multi-pinhole collimator (MILabs, Utrecht, Netherlands). SPECT data were acquired for 10 min at 1 and 6 h and 1 day; 15 min at 2 days; and 20 min at 3 days after the injection. SPECT images were reconstructed using a pixel-based ordered-subsets expectation maximization algorithm with 8 subsets and 2 iterations on a 0.8-mm voxel grid without attenuation correction. CT data were acquired using the X-ray source set at 60 kVp and 615 µA after SPECT scan, and the images were reconstructed using a filtered back-projection algorithm for cone beam. Merged images were obtained using PMOD software (PMOD Technology, Zürich, Switzerland). ROIs were manually drawn on tumours, and the mean and maximum values of the percentage of injected dose per gram of tissue (% ID/g) of ROIs was measured using PMOD software (PMOD Technology).

### Autoradiography

After the SPECT/CT imaging study, the mice were euthanized, and each tumour with brain was excised and frozen in Tissue-Tec optimal-cutting-temperature compound (Sakura Finetek). Frozen sections (20 µm thick) were fixed with 10% neutral buffered formalin (Wako), washed and dried. The dried sections were exposed to an imaging plate (Fuji Film, Tokyo, Japan), and the imaging plate was read using an FLA-7000 image plate reader (Fuji Film). After plate reading, the sections were stained with hematoxylin and eosin (HE). Tissue sections adjacent to those used for the autoradiographic study were immunostained with peroxidase-labelled anti-TF 1849 mAb. According to the manufacturer’s instructions, the peroxidase-labelled antibody was prepared using a peroxidase labelling kit (Dojindo, Kumamoto, Japan). The sections were fixed with 4% paraformaldehyde in PBS for 15 min at RT and endogenous peroxidase was blocked with 0.3% hydrogen peroxidase. After blocking with 5% skim milk in PBS overnight at 4 °C, the sections were incubated with peroxidase-labelled anti-TF 1849 mAb for 1 h at RT. The reaction was visualized by incubation with DAB (Dako), and hematoxylin was used for counter staining.

### Statistical analysis

The data for *ex vivo* fluorescence imaging and SPECT/CT imaging were analysed using the Mann-Whitney U test and repeated-measures ANOVA, respectively. All of the statistical tests were two-sided, and *P* < 0.05 was considered statistically significant. Statistical analyses were performed using SPSS Statistics Version 18 (SPSS, Chicago, IL, USA).
